# Intelligence, &c.

**Published:** 1829-04-01

**Authors:** 


### INTELLIGENCE, &c.

### ANOTHER PROVINCIAL JOURNAL.

We gladly announce the appearance of the " PROVINCIAL MEDICAL GAZETTE; or, South and West of England Hospital Reporter:" conducted by HENRY LYFORD, Esq. Surgeon to the Hampshire County Hospital. We welcome the stranger, and shall give every publicity to its contents. It is a quarterly journal, price 2s. 6d.

---

Mr. Wakley's " CASE OF REAL DISTRESS" is relieved—from the advertisement duty on nominal subscriptions.

---

We regret to inform our Caledonian subscribers to the fascicular form of this Journal, that Fasciculus II. of No. XX. was lost in the Leith packet, run down by the Lord Melville. The Fasciculus was instantly put to press, and we hope our subscribers will receive the second and third Fasciculi at the same time.

